# Fear of Falling, Recurrence of Falls, and Quality of Life in Patients with a Low Energy Fracture—Part II of an Observational Study

**DOI:** 10.3390/medicina57060584

**Published:** 2021-06-07

**Authors:** Puck C. R. van der Vet, Jip Q. Kusen, Manuela Rohner-Spengler, Björn-Christian Link, Roderick M. Houwert, Matthias Knobe, Reto Babst, Christoph Henzen, Lukas Schmid, Frank J. P. Beeres

**Affiliations:** 1 Department of Orthopaedic and Trauma Surgery, Luzerner Kantonsspital, 6000 Luzern, Switzerland; puck_vandervet94@hotmail.com (P.C.R.v.d.V.); jip.kusen@hotmail.com (J.Q.K.); manuela.rohner@luks.ch (M.R.-S.); bjoern-christian.link@luks.ch (B.-C.L.); Matthias.knobe@luks.ch (M.K.); reto.babst@luks.ch (R.B.); christoph.henzen@luks.ch (C.H.); lukas.schmid@luks.ch (L.S.); 2 Department of Trauma Surgery, University Medical Center Utrecht, 1084 CX Utrecht, The Netherlands; r.m.houwert@umcutrecht.nl; 3 Department of Health Sciences and Medicine, University of Lucerne, 6002 Luzern, Switzerland

**Keywords:** fear of falling, low energy fractures, fall prevention

## Abstract

*Background and objective*: Falls in elderly cause injury, mortality, and loss of independence, making Fear of Falling (FoF) a common health problem. FoF relates to activity restriction and increased fall risk. A voluntary intervention including fall risk assessment and prevention strategies was implemented to reduce falls in elderly patients with low energy fractures (LEF). The primary purpose of this study was to evaluate FoF and the number of subsequent falls in trauma patients one year after a LEF. The secondary aim was to examine how FoF affects patients’ lives in terms of Quality of Life (QoL), mobility, and activity levels. Finally, participation in the voluntary fall prevention program (FPP) was evaluated. *Materials and Methods*: Observational cohort study in one Swiss trauma center. LEF patients, treated between 2012 and 2015, were analyzed one year after injury. Primary outcomes were Falls-Efficacy Score-International (FES-I) and number of subsequent falls. Secondary outcomes were EuroQoL-5-Dimensions-3-Levels (EQ5D-3L), mobility, activity levels, and participation in the FPP. Subgroup analysis was performed for different age categories. *Results*: 411 patients were included for analysis. Mean age was 72 ± 9.3, mean FES-I was 21.1 ± 7.7. Forty percent experienced FoF. A significant negative correlation between FoF and QoL (R = 0.64; *p* < 0.001) was found. High FoF correlated with lower activity levels (R= −0.288; *p* < 0.001). Six percent visited the FPP. *Conclusions*: At follow-up, 40% suffered from FoF which seems to negatively affect patients’ QoL. Nevertheless, participation in the FPP was low. Simply informing patients about their susceptibility to falls and recommending participation in FPPs seems insufficient to motivate and recruit patients into FPPs. We suggest implementing repeated fall risk- and FoF screenings as standard procedures in the follow-up of LEF, especially in patients aged over 75 years.

## 1. Introduction

The prevalence of falls in the elderly is high and the incidence increases exponentially with age [1]. Annually, one in three patients aged 65 years or older experience one or more falls [2,3]. The consequences in frail elderly are often severe, many resulting in a fracture. The physical, social, and psychological outcomes after a fracture in elderly patients are known to be poor and a fall itself is an independent risk factor for a subsequent fall [4]. The fall-related mortality rates are high and increase with age [5,6,7]. However, early identification of fallers using multiple measurement sets shows shortcomings. Sensitivity and specificity of the commonly used mobility and balance indices are moderate or poor [8]. Therefore, fear of falling (FoF) amongst elderly is a useful evaluation tool but moreover a common health problem. Its prevalence is estimated to be between 20 and 55% [5]. Previous literature reports that FoF negatively affects recovery after a fracture [5,9]. Furthermore, high FoF has been associated with the occurrence of (secondary) fractures, poorer Quality of Life (QoL) outcomes, and lower activity levels, all of which have economic consequences [6,10,11,12]. FoF potentially causes total avoidance of activities, loss of functional independence, and reduction in social activities [12,13,14]. FoF exists not only in patients who already experienced falls, but is also present in up to 35% of patients who have never experienced a fall [10,15]. With the aging population, the number of falls will continue to increase and efficient fall prevention strategies are needed.

We studied a strategy that was implemented to reduce the recurrence of falls and fractures in patients aged between 50–85 years and who were referred to the emergency unit for a low energy fracture (LEF). LEF was defined as: ‘a fracture resulting from a fall from standing height or less’ [16]. The prevention strategy was based on the implementation of dedicated health professionals (HPs) who were trained to inform patients on fall risks and osteoporosis and who aimed to motivate patients to participate in a fall prevention program (FPP).

We hypothesized that FoF would still be present one year after LEF and that it would be higher in patients who had at least one subsequent fall or those with a secondary fracture [17]. Furthermore, we believed that there would be a relation between FoF and QoL.

## 2. Materials and Methods

### 2.1. Design

Single-center cohort study. This study is written in accordance with the STROBE-criteria [18].

### 2.2. Participants, Center

Patients who were treated for LEF between May 2012 and December 2015 and who met all inclusion criteria were eligible for inclusion. Inclusion criteria can be found in the previously published study [19]. One year after LEF, all eligible patients were sent a study invitation. Along with the study information, patients received an informed consent form and a structured questionnaire, both to be completed and returned to the study coordinator. All patients who returned the completed study documents and therefore were willing to participate in the study were included. This study was the second part of a research project on a secondary prevention program for patients with LEF called ‘Osteofit’ [19]. The first study on this project focused on screening and prevention of osteoporosis. Eligibility criteria, patient flow chart, and patient characteristics can be found in the abovementioned study.

### 2.3. Secondary Fall Prevention Intervention

To improve secondary prevention of falls and fractures after primary LEF, dedicated HPs were implemented. The tasks of the dedicated HPs were (1) to screen the patient list of the emergency unit for fracture patients equal or older than 50 years, (2) to screen the electronic medical records (EMR) of these preselected patients, and (3) to include all patients with a report of LEF into the secondary FPP. Thereafter, the HPs were assigned to (1) inform the patients about their susceptibility to subsequent falls and fractures and (2) to motivate them to visit the dedicated physiotherapist with specific skills in fall prevention. Patients were informed about the FPP in two ways. First, inpatients were personally informed during their hospital stay by the dedicated HPs. Second, outpatients or patients who could not be reached during their hospital stay were contacted and informed by an informative letter and by telephone. The patients who visited the dedicated physiotherapist received a comprehensive fall risk assessment and individual recommendations for fall prevention. Fall risk assessment included the evaluation of intrinsic and extrinsic fall risk factors (co-morbidities, medication, falls history, nutrition, mobility, cognition, vision, living situation, etc.) and the following physical assessments: timed-up and go test, five chair rise, balance (assessed according to the 3 balance items of the Short Physical Performance Battery with side-by-side stand, semi-tandem stand, tandem stand), and with single leg stand) and gait velocity [20].

### 2.4. Data Collection

Data collection started in 2013. Baseline data at time of injury and follow-up data after a one-year follow-period were collected. Age, gender, body mass index (BMI), and American Society of Anesthesiologists (ASA) scores were obtained through the patients′ EMR. The fractures were grouped based on anatomic localization (humerus, clavicle, rib, radius/ulna, femur, tibia, fibula, patella, malleolar segment, spine, pelvic ring, acetabulum, hand, foot, or craniomaxillofacial bones). For the collection of one-year follow-up data, patients received both a standardized, multiple-choice questionnaire by mail and a telephone interview, consisting of the validated instruments to assess FoF and QoL (see below) and of structured questions with multiple-choice answers (Appendix A). To improve data quality, some questions in the interview and questionnaire were redundant.

*Primary outcomes:* FoF (assessed through both the Falls-Efficacy Score-International (FES-I) and a binary patient-reported outcome) and the number of subsequent falls and fractures. The FES-I has scores rating from 16 (lowest) to 64 (highest). In addition, the FES-I score was subcategorized into three categories, based on previous literature: ‘Low’ (FES-I score of 16–19), ‘medium’ (FES-I score of 20–27), and ‘high’ (FES-I score of 28–64). (8, 16).

*Secondary outcomes:* QoL (measured by the EuroQol-5-Dimensions-3-Levels (EQ5D-3L)), mobility, hours of outside activity, and participation rate in the fall prevention program.

The EQ-5D-3L exists of a questionnaire and a visual analogue scale (VAS), with scores varying from 0 to 1.0 (0 being the lowest possible QoL score and 1 being the highest QoL score). Postoperative mobility was scored into ‘ambulant’ if the patient could walk independently or ‘mobility accessories’ if the patient used one or two walking aids (e.g., walkers, crutches, canes). Hours of outdoor activity was categorized into: ‘less than 20 or 30 min per day’, ‘more than 20–30 min per day’, ‘more than 1 h per day’, and ‘more than 2 h per day’. Subgroup analysis were made according to different FoF-and age categories, namely patients aged 65–74 years of age and patients aged 75 years or older.

### 2.5. Data Analysis

Results were evaluated descriptively, using total numbers and percentages for categorical variables. Mean values (M) along with the standard deviations (SD) were used for numeric, normally distributed data. Medians and interquartile ranges (IQR) were calculated for numeric, skewed data. For QoL (nonparametric), both the median and mean were calculated in order to compare to previous literature. The Shapiro-Wilks test was used to assess normal distribution. Missing values were assessed through available case analyses and we used the Spearman test to assess potential correlations. In addition, we used partial correlations. We used the X2-test to assess differences in categorical values. *p*-values with a significance level of 0.05 were considered statistically significant. The Mann-Whitney U-test or the Kruskal-Wallis test were used for continuous non-parametric data and the 95% confidence intervals (CIs) and median differences (mdn diff) were calculated using the Hodges-Lehmann test. For parametric data, the independent samples T-test was used for the 95% CI and the mean difference (mean diff.). Data were analyzed using SPSS Statistics version 25 (IBM Corporation Armonk, NY, USA). Baseline data of non-participants was compared to baseline data of participants.

## 3. Results

### 3.1. Flow of Participants and Baseline Characteristics

As mentioned, this study was part of a larger research project. Flow of participants and baseline characteristics have been described in the previously mentioned associated study [19]. Response rate of this study was 50% (411 of 823 patients).

### 3.2. Prevalence of FoF and Subsequent Falls and Fractures

Table 1 shows postoperative outcomes in all patients and Table 2 shows postoperative outcomes according to FoF categories. The mean FES-I score of all patients was 21.1 ± 7.7 and did not differ between men and women (M♂20.6 ± 8.2 vs. M♀21.2 ± 7.5; *p* = 0.497). Sixty patients (15.5%) experienced one or more falls after one year and fifteen patients experienced a subsequent fracture (4.2%), ten of which expressed FoF. Mean FES-I score of the patients with a subsequent fracture was 25.3 ± 9.2 as compared to patients without a subsequent fracture who had a mean FES-I score of 21 ± 7.5 (Mean difference: 4.26; *p* = 0.03; 95%CI 0.33–8.2). In patients who did not fall in the one-year follow-up period mean FES-I score was 20.7 ± 7.1, whereas it was 23.7 ± 9.8 in patients who did have a subsequent fall (Mean Difference 2.99; *p* = 0.006; 95% CI 0.88–5.01). Moderate to high FoF scores, as measured with the FES I, was found in 161 patients (39.6%) and FoF as measured as a binary outcome with the simple question ‘are you afraid of falling’ was answered with ‘yes’ in 152 of all patients (40.2%). In patients who experienced a subsequent fall, 67% reported to be afraid of falling. Between FoF categories, there was a significant difference in age, pre-and post-injury activity hours, rehospitalization, and subsequent fractures (Table 2).

### 3.3. Effect of FoF on QoL, Mobility, and Activity Levels

Median QoL index score in all patients was 0.9 (0.75–1.0) and mean QoL index score in all patients was 0.86 ± 0.20, median QoL VAS score was 90 (71.3–95), and mean QoL VAS score 82.1 ± 17.2. In patients with high FoF (FES > 27), QoL was significantly lower than in patients with low to moderate FoF scores (0.61(0.29–0.74) versus 1.0 (0.85–1.0), Median difference 0.34; *p* < 0.001; 95%CI 0.28–0.39). QoL VAS was also significantly lower in patients with high FoF (Median difference 30; *p* < 0.001; 95%CI 25–33). In patients without a subsequent fall it was 0.95 (0.76–1.0), whereas it was 0.79 (0.70–1.0) in patients with a fall (*p* < −0.001). Overall, there was a significant negative correlation between FoF and QoL at a moderate level (R = −0.64; *p* < 0.001). FoF also correlated with age (R = 0.252, *p* < 0.001) a higher number of subsequent falls (R = 0.127, *p* = 0.006) and a lower activity level (R = −0.288, *p* < 0.001). After injury, 38.8% of the patients with high FES I scores (FES I > 27) were active for less than 20–30 min a day outside compared to patients with FES I scores lower than 19, 10.6% reported to be active less than 20 min (*p* < 0.001). At follow-up, 99.4% of patients were still ambulatory (353 out of 355 patients) and the number of patients using one or more walking aid devices was 2 (0.6%).

### 3.4. Participation Rate at the FPP

In total, 21 patients (6%) visited the dedicated physiotherapist for fall risk assessment and fall prevention. Of these 21 patients, 15 (71.4%) were reached and informed about the FPP by the dedicated health professional during their inpatient treatment. The remaining 6 patients received outpatient treatment and were informed about their potential risk of falling and the recommendation to the FPP by mail and telephone. Mean age was 75.1 ± 6.7, 90.5% were female, mean FES I score was 22.1 ± 6.5. Mean QoL index score was 0.76 ± 0.28 and QoL VAS was 78.5 ± 22.2. There were no significant differences between visitors of the FPP and non-visitors in age (*p* = 0.182), gender (*p* = 0.393), FES-I score (*p* = 0.492), QoL index (*p* = 0.129), and QoL VAS score (*p* = 0.421) Reasons for not participating in the FFP were: patients did not think it was necessary (14.8%), no time (1.4%), too much effort (5.1%), other reasons (18.8%), and 39.9% of the patients were unaware that it was recommended to them.

### 3.5. Partial Correlations

#### 3.5.1. FoF and Post Injury Hours of Activity

A small, negative partial correlation between FoF and post injury hours of activity was found when controlling for age, gender, and QoL which was statistically significant (R) (361) = −0.114, *n* = 353, *p* = 0.033). Zero-order correlation revealed a correlation of R (351) = −0.305, *n* = 353, *p* < 0.001) indicating that age, gender, and QoL had an influence in controlling for the relationship between FoF and post injury hours of activity.

#### 3.5.2. FoF and QoL

The partial correlation between FoF and QoL, controlling for age, gender, post injury hours of activity, and subsequent falls revealed a moderate inverse correlation (R (151) = −0.641, *n* = 157, *p* < 0.001).

#### 3.5.3. FoF and Number of Subsequent Falls

After controlling for QoL, age, gender, and postinjury hours of activity, the partial correlation between FoF and subsequent falls was R (344) = −0.51, *n* = 353, *p* = 0.353. Zero-order correlation showed a correlation of (R (348) = 0.127, *n* = 353, *p* = 0.018), indicating that the other factors had a significant influence in controlling for the relationship between FoF and subsequent falls.

#### 3.5.4. FoF and Age

A partial correlation of R (344) = 0.020, *n* = 350, *p* = 0.713 was found between FoF and age when controlling for gender, QoL, postinjury hours of activity, and subsequent falls.

### 3.6. Subgroup Analysis

#### 3.6.1. Patients Aged 65–74 Years

Mean FES-I score was 19.3 ± 5.1. In total, 9 patients had high FoF (6.8%) and 15 patients (11.9%) experienced a subsequent fall. Median QoL index score was 1.0 and median QoL VAS score was 90. Mean QoL index score was 0.90 and mean QoL VAS score was 85.1.

#### 3.6.2. Patients Aged ≥75 Years

Mean FES-I score was 22.9 ± 8.9. A total of 33 patients experienced a high level of FoF (18.6%) and 33 patients experienced a subsequent fall (18.9%). Median QoL index score was 0.9 and median QoL VAS score was 80. Mean QoL index score was 0.81 and mean QoL VAS score was 77.5.

Between these two age subgroups, a significant difference was found in FES-I score (19.3 vs. 22.9; mean diff: 3.5; 95%CI 1.9–5.2; *p* < 0.001) and in QoL index score (1.0 vs. 0.9; median diff: 0; 95%CI 0.00–0.10; *p* < 0.001) and QoL VAS score (90 vs. 80; median diff: 5; 95%CI 5–10; *p* < 0.001). Additionally, significant differences were found in postoperative hours of activity (*p* < 0.001). No significant differences were found in postoperative mobility and visits to the FPP. Details are shown in Table 3.

## 4. Discussion

In this study we observed that one year after LEF in a population of mostly ambulatory, community-dwelling elderly adults, the prevalence of FoF was 40%. In patients over 75 years of age, in recurrent fallers, and in patients who experienced a subsequent fracture in the follow-up period, FoF was even more accentuated and FES-I scores were significantly higher. In patients with high expressions of FoF, QoL and activity level were significantly lower and a significant negative correlation at a moderate level was found between FoF and QoL. Furthermore, FoF correlated with a lower activity level. The fall prevention intervention that was recommended and offered to all patients was poorly visited (6%).

Previous literature on FoF in elderly trauma patients reports that the prevalence of FoF in community-dwelling elderly ranges between 22 and 55% and that FoF is present in patients who did and did not experience a fall [5,21,22]. Cumming et al. investigated FoF in 418 community-dwelling patients of 65 years of age or older and found a prevalence of 30% (as measured by an answer to a binary question). Comparable to our results, they also reported that FoF negatively affects recovery in terms of mobility, QoL and activity level after a fracture [23]. The higher prevalence of FoF in our study may be attributed to the fact that we investigated patients who already experienced a fracture and its consequences. Another study from England, which included 2212 community-dwelling adults between 65 and 84 years old, found that the prevalence of FoF after a 20 month follow-up period was 23.2%. The authors reported FoF to be an independent risk factor for experiencing a subsequent fall. In contrast to our study, 72.2% of their study participants did not have a history of falls at baseline (e.g., in the previous 12 months) and FoF in their study was assessed both at baseline and at follow up, enabling the investigation of the causality of FoF and subsequent falls. Their findings support our hypothesis that higher FES-I scores in patients who had a subsequent fall, as compared to the ones who did not fall, may be both a result and a cause of the subsequent fall in the one year of follow up, therefore the correlation between FoF and a secondary fall or fracture could be regarded as a vicious circle.

Even though we found a high prevalence of FoF and its negative consequences, adherence to the recommendation to visit the fall prevention specialist for a comprehensive fall risk assessment was very low (6%), also in patients aged 75 years or older (7.5%). Amacher et al. and Child et al. came to the same conclusions and found that the main reasons for not adhering to the recommendations were: costs of the FPP, transportation, shortage of time, unwillingness to participate, and unable to visit the hospital [24,25]. Mihaljcic et al., 2015 concluded that elderly undergoing inpatient rehabilitation underestimate their personal fall risk. Based on their findings in 2017 they suggested self-awareness of fall risk to be associated with rehabilitation engagement and motivation and concluded that improving patient self-awareness of fall risk may increase engagement in therapy. Hill et al., 2014 added that improved communication and education strategies and engaging family may also increase adherence. Mikolaizak et al., 2017 found that both high baseline intention to adhere and a higher number (>3) of recommendations prescribed were the most important factors that determine adherence to the multifactorial fall-prevention intervention [26,27,28,29,30]. Elley et al. investigated the effectiveness of a community-based, nurse-led multifactorial falls and fracture prevention intervention. They concluded that implementation and adherence was dependent on referral to other health professionals and that this may have limited the effectiveness of the interventions. In this study, most of the FPP non-participants (39.9%) did not seem to be aware that they were recommended to visit the HP for fall prevention. One reason for this finding may be that the dedicated HPs focus and the information letter were more accentuated on the prevention of osteoporosis than on fall prevention. Another reason could be that the patients neglected the recommendation due to, as discussed above, lack of self-awareness. It is also notable that patients who were visited by the HP in person were more willing to participate than patient who were contacted by phone. Personal contact in recruiting patients for FPP may increase patients’ willingness to participate. Additionally, recent literature shows the additional value of using 3D models to improve understanding of the healed fracture site, possible underlying disease (i.e., osteoporosis), confidence in training and therefore willingness to participate in FPPs. 3D models can also be used to improve the patient–doctor relationship [31].

Various mono- and multifactorial interventions have been proven effective in reducing fall risk and FoF in different populations and lots of efforts have been undertaken by health care providers to build-up comprehensive FPPs and facilitate access [32,33,34]. Nevertheless, as discussed above and observed in this study, recruiting patients into such programs and adherence to recommendations remains a major challenge. Perhaps it would be advantageous to pretest at-risk individuals in their own environment using simple self-assessment tools, with direct guidance by the general practitioner (GP) as the main contact person in fall risk evaluation and fall prevention. With this approach, cost savings in the healthcare system are possible, combined with a higher health-related QoL in the geriatric population [35]. This potential of a predominant role of the GP in fall prevention was described by other authors and should be advocated [36].

This study has a number of limitations. First, we sent out study invitations one year after trauma, which caused patients to answer some questions from memory. This may have led to recall bias and an underestimation in the number of subsequent falls [37]. Nevertheless, considering the binary question whether they had had at least one subsequent fall, we believe the results to be rather robust. To minimize interviewer bias, we used validated instruments and structured multiple-choice questionnaires. Secondly, we did not have FES-I scores of the patients at baseline. Consequently, we cannot make any conclusions about the causality of FoF on subsequent falls and fractures. Based on previous literature, we consider our 50% response rate to be acceptable [7,21,22,25]. To control for a potential selection bias, comparison of relevant baseline data was performed and revealed that non-participants were comparable to participants considering age, gender distribution, fracture type and treatment setting (*p* > 0.05). Therefore, the risk of selection bias is minor. Third, considering generalization, it should be noted that the patients included in this study were younger than in others and mostly community-dwelling. This was a deliberate decision, because we aimed to study specifically those patients in which a secondary FPP, consisting of fall prevention and the prevention of osteoporosis, would be most beneficial. Additionally, to minimize this bias, a subgroup analysis was made according to the different age categories. Last, the number of FPP participants was small compared to the whole study population (21 vs. 390), which makes it difficult to draw conclusions and limits conclusiveness.

## 5. Conclusions

To our knowledge, this study is the first evaluating FoF in relation to subsequent falls, subsequent fractures, and to QoL in a cohort of LEF patients one year after trauma. In all age categories, but particularly in patients over 75 years of age, the prevalence of FoF is high and negatively affects the patients′ QoL and activity level. Despite this finding, participation in a voluntary FPP in this group was very low and unsatisfactory. Simply informing patients about their potential risk of falling and advising them to have their fall risk assessed does not seem to be sufficient to encourage and recruit LEF patients into a FPP. Hence, more efforts need to be undertaken. The implementation of simple fall risk assessments taken directly in the emergency or trauma unit or simple self-administered fall risk assessments may could help to improve patients′ self-awareness of fall risk and may be an option to recruit more patients into a comprehensive FPP.

Furthermore, we recommend to not only incorporate physical but also psychological measures such as FoF into the follow up of elderly LEF patients. Patients screened at risk for falling or with high expressions of FoF need to be prescribed and referred to adequate fall preventive interventions.

Lastly, to further improve self-awareness of fall risk, repeated screenings should be performed on a regular basis throughout the rehabilitation process and thereafter. Involvement of the GP to guide the patients and promote fall prevention is strongly recommended.

## Figures and Tables

**Table 1:** Postoperative outcomes.

Fear of Falling In	
All patients mean ± SD	21.1 ± 7.7
Patients with ≥1 subsequent fall mean ± SD	23.7 ± 9.8
Patients without subsequent fall mean ± SD	20.7 ± 7.1
Patients with a subsequent fracture mean ± SD	25.3 ± 9.2
Patients aged 50–69 years mean ± SD	19.9 ± 6.9
Patients aged 70–79 years mean ± SD	20.6 ± 6.4
Patients aged 80 plus year mean ± SD	23.3 ± 9.5
Fear of Falling categorized:	
Low (FES-I score: 16–19) *n* (%)	245 (60.3)
Moderate (FES-I score: 20–27) *n* (%)	109 (26.8)
High (FES-I score: 28–64) *n* (%)	52 (12.8)
Subsequent Falls:	
0 falls *n* (%)	326 (84.5)
1 fall *n* (%)	44 (11.4)
2 falls *n* (%)	9 (2.3)
3 falls *n* (%)	3 (0.8)
4 falls *n* (%)	4 (1.0)
Hours of daily outdoor activity:	
<20–30 min *n* (%)	62 (16.8)
≥30 min *n* (%)	127 (34.4)
>1 h *n* (%)	131 (35.5)
>2 h *n* (%)	49 (13.3)
Postoperative mobility:	
Ambulant *n* (%)	353 (99.4)
Mobility accessories *n* (%)	2 (0.6)
Visits to the FFP	
Yes *n* (%)	21 (6)
No *n* (%)	330 (94)

*n* (%): absolute and relative values of the proportion of analyzed study patients. *SD*: Standard Deviation. FES: Falls Efficacy Score. Missing data was excluded from analysis.

**Table 2:** Postoperative outcomes categorized by FoF category.

	All Patients (*n* = 411)	Low FoF (*n* = 245)	Medium FoF (*n* = 109)	High FoF (*n* = 52)	*p*-Value
Age mean *SD*	72.0 (9.3)	70.3 (9.0)	74.3 (8.9)	74.9 (9.2)	**<0.001**
Male *n* (%)	83 (20.2)	55 (22.4)	17 (15.6)	10 (19.2)	0.328
Preinjury activity hours *n* (%)					**<0.001**
<20–30 min	33 (19.0)	8 (9.2)	26 (27.1)	8 (32.0)	
≥30 min	55 (31.6)	22 (25.3)	19 (32.2)	13 (52.0)	
>1 h	75 (43.1)	47 (54.0)	23 (39.0)	4 (16.0)	
>2 h	11 (6.3)	10 (11.5)	1 (1.7)	0 (0.0)	
Post injury activity hours *n* (%)					**<0.001**
<20–30 min	62 (16.8)	23 (10.6)	19 (19.2)	19 (38.8)	
≥30 min	127 (34.4)	65 (30.0)	44 (44.4)	17 (34.7)	
>1 h	131 (35.5)	92 (42.2)	29 (29.3)	8 (16.3)	
>2 h	49 (13.3)	37 (17.1)	7 (7.1)	5 (10.2)	
Subsequent falls *n* (%)					0.188
0 falls	326 (84.5)	196 (86.3)	90 (87.4)	36 (70.6)	
1 fall	44 (11.4)	23 (10.1)	11 (10.7)	10 (19.6)	
2 falls	9 (2.3)	4 (1.8)	2 (1.9)	3 (5.9)	
>2 falls	7 (1.8)	4 (1.8)	0 (0)	2 (4.0)	
Rehospitalization	12 (3.1)	3 (1.3)	3 (2.9)	6 (11.8)	**<0.001**
Subsequent fractures *n* (%)	15 (3.9)	6 (2.6)	3 (2.9)	6 (11.8)	**0.008**
Visit FPP *n* (%)	21 (6.0)	9 (4.3)	7 (7.6)	4 (9.3)	0.296

*n* (%): absolute and relative values of the proportion of analyzed study patients. *SD*: Standard Deviation. FoF: Fear of Falling, low: Falls Efficacy Score 16–19, medium 20–27, high >27. FPP: fall prevention program. The bold number shows that the result is statistically significant.

**Table 3:** Subgroup analysis according to different age categories.

All Patients Aged ≥65 Years, According to Different Age Categories				
		Patients Aged 65–74 Years (*n* = 139)	Patients Aged ≥ 75 Years (*n* = 179)	*p*-Value
FoF mean ± *SD*	All patients within the age category	19.3 ± 5.1	22.9 ± 8.9	**<0.001**
	Patients with <1 subsequent fall	19.5 ± 5.1	21.9 ± 7.9	**<0.001**
	Patients with ≥1 subsequent fall	19.2 ± 6.8	26.3 ± 10.7	**0.047**
FoF categorized *n* (%)	Categorized: Low	96 (69.6)	84 (47.5)	**<0.001**
	Categorized: Moderate	33 (23.9)	60 (33.9)	
	Categorized: high	9 (6.5)	33 (18.6)	
Binomial *n* (%)	FoF	45 (35.5)	48 (47.3)	**0.042**
	No FoF	82 (64.6)	87 (52.7)	
Secondary Falls *n* (%)	0 falls	111 (88.1)	142 (81.1)	0.500
	1 fall	11 (8.7)	22 (12.6)	
	>1 fall	4 (3.2)	11 (6.3)	
Hours of Daily Outdoor Activity *n* (%)	<20–30 min	16 (13.1)	40 (23.7)	**<0.001**
	>30 min	31 (25.4)	73 (43.2)	
	>1 h	58 (47.5)	44 (26.0)	
	>2 h	17 (13.9)	12 (7.1)	
Postoperative mobility *n* (%)	Ambulant	123 (99.2)	150 (99.3)	0.889
	Mobility accessory	1 (0.8)	1 (0.7)	
QoL *median* (*IQR*)	EQ-5D-3L index	1.0 (0.85–1.0)	0.9 (0.74–1.0)	**<0.001**
	EQ-5D-3L VAS	90 (80–95)	80 (70–90)	**<0.001**
Visits to the Osteofit consult *n* (%)	Yes	8 (6.8)	12 (7.5)	0.802

*n* (%) absolute and relative values of the proportion of analyzed study patients. *SD*: Standard Deviation. Q-5D-3L: EuroQol-5-Dimension-3-Level score. Missing data was excluded from analysis. VAS: visual analogue scale. The bold number show a statistically significant result.

## Data Availability

The data presented in this study are openly available through the website of Medicina. Previously reported data on baseline characteristics is available from: https://dx.doi.org/10.1007/s11657-019-0595-0.

## Appendix A

One-year follow-up written questionnaires (in English, FES-I and EQ-5D-3L excluded).
